# Professional growth in pharmacy: examining CPD awareness, motivators, and barriers among pharmacists

**DOI:** 10.1080/20523211.2025.2490985

**Published:** 2025-05-27

**Authors:** Lubna Qaisi, Eman Alefishat, Rana Abu Farha, Amal Akour, Mohammad Zawieh

**Affiliations:** a Faculty of Pharmacy, The University of Jordan, Amman, Jordan; b Department of Medical Sciences, College of Medicine and Health Science, Khalifa University of Science and Technology, Abu Dhabi, UAE; c Department of Biopharmaceutics and Clinical Pharmacy, School of Pharmacy, The University of Jordan, Amman, Jordan; d Center for Biotechnology, Khalifa University of Science and Technology, Abu Dhabi, UAE; e Department of Clinical Pharmacy and Therapeutics, Faculty of Pharmacy, Applied Science Private University, Amman, Jordan; f Department of Pharmacology and Therapeutics, College of Medicine and Health Sciences, United Arab Emirates University, Al-Ain, UAE; g Department of Clinical Practice, College of Pharmacy, Northern Border University, Rafha, Saudi Arabia; h Department of Pharmacy Practice, College of Clinical Pharmacy, Hodeidah University, Hodeidah, Yemen

**Keywords:** Continuous professional development, pharmacists, awareness, views, Jordan

## Abstract

**Background:**

In the evolving landscape of pharmacy, the shift towards patient-centred care necessitates continuous professional development (CPD) for pharmacists. This study aims to assess the awareness, perception, motivators, and barriers to CPD implementation among pharmacists.

**Methods:**

A cross-sectional study was conducted, utilising a structured questionnaire. Convenience sampling was employed, inviting 250 pharmacists from diverse practice settings to participate. Descriptive statistics were used for analysis.

**Results:**

Out of the 210 pharmacists who participated in the study, more than half (51.7%) were unfamiliar with CPD, while only a small percentage (3.8%) were very familiar with it. Engagement in learning activities was common, reported by 80% of participants, with varying frequencies: weekly (19%), monthly (30.5%), yearly (27.6%), and rarely (22.9%). However, the implementation of CPD cycle elements was minimal, with only 8.6% reflecting, 5.7% planning, 10.5% taking action, and 7.2% evaluating their learning activities more than 75% of the time. Most pharmacists supported mandatory CPD (80.6%) and integration into a legal framework (81.3%). Anticipated benefits, such as keeping knowledge updated (92.9%) and improving patient care (91%), were highly endorsed. Motivators for CPD included proximity to the workplace (82.3%) and flexible schedules (84.7%), while barriers included time constraints (73.7%) and lack of information (74.2%).

**Conclusion:**

Pharmacists demonstrate low awareness and implementation of CPD, underscoring the necessity for educational initiatives and legislative support. Motivators such as proximity and flexible schedules could enhance CPD uptake, while addressing barriers like time constraints and lack of information is crucial for successful CPD integration in pharmacy practice.

## Background

In the area of patient-centred care, pharmacy has significantly shifted from traditional practices of compounding and dispensing of medicines (Adams & Weaver, 2021). Presently, pharmacy has evolved into a patient facing profession with broader and a greater focus on patient care (Adams & Weaver, 2021). Most notably, pharmacists now bear primarily responsibility for counselling patients on the safe use of medications, providing education regarding potential side effects, and conducting reviewing any possible drug–drug interactions. These efforts contribute significantly to improved health-care outcomes (WHO, 1998; Zerrin Toklu, 2013).

Given the expanding roles and responsibilities, pharmacists are faced with the challenge of keeping up to date with the ongoing advancements in medical sciences and health care. Due to the complex nature of pharmacy as a profession and rapidly changing medical and technological aspects, lifelong learning of pharmacists is a key in keeping them competent. Continuing Education (CE) had been used in multiple disciplines of medicine and found to be an efficient and effective learning method with decent improvement of learning outcomes. However, when implemented in pharmacy practice, CE has not been consistently resulted in the anticipated changes in pharmacists’ attitude and behaviour (Micallef & Kayyali, 2019).

One limitation of CE programs, contributing to their ineffectiveness, is a lack of focus on specialty-oriented topics. This results in decreased interest among the pharmacists in the topics offered. Therefore, there is an urgent need to customise CE to align with the needs and interests of today's pharmacists (Driesen et al., 2007; Rouse, 2004a). Continuous Professional Development (CPD) offers a self-directed, outcome-oriented approach that enables pharmacists to engage in activities tailored to address their individual needs, and align with their various career goals, which have a stronger positive influence on pharmacists’ professional attitude and performance (Batista et al., 2022).

The International Pharmaceutical Federation (FIP) defined CPD as ‘the responsibility of individual pharmacists for systematic maintenance, development and broadening of knowledge, skills and attitudes, to ensure continuing competence as a professional, throughout their careers’ (FIP, 2002). This is achieved by a series of actions, including self-assessment, planning, implementation, evaluation, and recording. These actions are encapsulated in what is termed the CPD cycle, comprising four major steps: reflection (to reflect on a learning goal or objective based on personal interests), planning (forming a plan with the activities to be implemented with a realistic deadline for the activities to be established), action (to perform the learning activities) and evaluation (to evaluate learning activities accomplishments). Evaluation at the end of the current year's cycle will serve as a milestone in planning for the year after (FIP, 2002; Tran et al., 2014). CPD appears to offer pharmacists a viable model for customised lifelong learning and personal improvement. It has been easily adopted and well accepted among pharmacists, leading to significant improvements in behavior and attitudes when implemented in multiple countries (Attewell et al., 2005; McConnell et al., 2010; Power et al., 2008; Rouse, 2004b).

In Jordan, pharmacists frequently engage with patients in various encounters; often serving as primary source of consultation for non-urgent health issues and drug related questions in both formal and informal settings (e.g. during social interactions) (Barakat et al., 2021; Wazaify et al., 2008). The implementation of the CPD approach by Jordanian pharmacists has the potential to enhance their competency, clinical skills, positive attitudes, excellence, and their reputation among other health-care professionals as trustworthy health-care providers. However, despite these potential benefits, neither the Ministry of Health (MOH) nor the Jordanian Pharmacists Association (JPA) mandates or offers continuous education programs to engage pharmacists in CPD activities.

Currently, there is limited data regarding awareness, perception, and implementation of CPD approach among Jordanian pharmacists. To address this gap, we developed a structured questionnaire aimed at assessing the present status, perspectives, motivators, and barriers for the implementation of CPD among Jordanian pharmacists.

## Methods

### Study design, settings, and subjects

This cross-sectional study was designed to evaluate the current status, perceptions, attitudes, motivators, and barriers to the implementation of CPD among pharmacists in Jordan. The study was conducted in Amman, Jordan, between August and September 2017. Using a convenience sampling method, 250 practicing pharmacists from various practice settings (private hospitals, community pharmacies, academia, industry, and drug companies) were invited to participate and asked to complete a paper-based survey. Pharmacists registered with the Jordan Pharmacists Association were eligible for inclusion, and no exclusions were applied.

### Questionnaire development

A comprehensive literature review of different electronic databases (MEDLINE/PUBMED, OVID, and EMBASE) was performed to identify relevant published English-language literature about pharmacist knowledge, attitude and practice regarding the CPD approach. The search process was conducted using the following keywords: ‘pharmacists’, ‘knowledge’, ‘attitude’, ‘practice’, and ‘continuous professional development’ and around ten studies were found to be related. Based on the results of the reviewed literature, a questionnaire was built to evaluate the aim of this study (Bellanger & Shank, 2010; Dopp et al., 2010; USAID, 2013). Pilot testing of the final draft of the questionnaire was performed by distributing the draft to ten pharmacists selected using a convenience sampling technique. The feedback from those 10 pharmacists was used to modify the original questionnaire questions and make them clearer and more comprehensible.

The final questionnaire was organised into several sections. The first section gathered information about the pharmacists’ demographics. The second section focused on their awareness of CPD. The third section explored their perceptions of how CPD is implemented, while the fourth section delved into their views on the benefits of CPD. The fifth section highlighted the factors that motivate pharmacists to participate in CPD activities, and the sixth section identified the barriers they face. Finally, the last section examined their recent experiences with CPD activities. Responses to Sections 3 through 6 were measured using a 5-point Likert scale, ranging from ‘strongly agree’ to ‘strongly disagree’. The survey was conducted in English, as it is the language of pharmacy education in Jordan.

Before approving the study questionnaire, pilot testing was conducted with a group of community pharmacists to assess the reliability and internal consistency of the instrument. The calculated Cronbach's alpha values for sections 3, 4, 5, and 6 were 0.818, 0.915, 0.929, and 0.658, respectively, indicating acceptable reliability. The data collected during the pilot testing were excluded from the final analysis.

### Ethical consideration

The study was approved by the Institutional Review Board (IRB) at Jordan University Hospital (IRB number: 10/2017/1348). Written informed consent was obtained from all participants before their involvement in the study, ensuring that participation was voluntary and that all responses were kept anonymous.

### Sample size calculation

For the questionnaire, sample size was calculated based on O'Rourke et al. (2013), where it is recommended that the number of subjects should be at least 5 times the number of items or 100. Given that we have 42 items in our questionnaire, a minimal sample size of 210 pharmacists was considered representative for the purpose of this study.

### Statistical analysis

Data were analysed using statistical package for social science (SPSS®) version 22 (SPSS® Inc., Chicago, IL, USA). The descriptive analysis was done using median and interquartile range (IQR) for continuous variables and frequency/percentage for categorical variables.

## Results

We distributed the survey to 250 pharmacists until we received 210 responses, achieving a response rate of 84%. The majority of participating pharmacists were young, with a median age of 28.0 years (IQR = 6.0). A significant proportion were female (*n* = 164, 78.1%), with a median work experience of 4.0 years (IQR = 7.0). Most pharmacists held a Bachelor's degree (BSc or PharmD) (*n* = 177, 84.3%), and nearly half of them worked in community pharmacies (*n* = 104, 49.5%). Table 1 provides further details on the demographic characteristics of the study participants.

**Table 1.** Demographic characteristics of the study sample (*n* = 210).

Parameter	Median (IQR)	*n* (%)
Age (years)	28.0 (6.0)	
Working experience	4.0 (7.0)	
Gender o Male o Female		46 (21.9) 164 (78.1)
Academic degree o Bachelor's degree (BPharm/PharmD) o Post-graduate degree (MSc/PhD)		177 (84.3) 33 (15.7)
Working Area o Community pharmacy o Private hospital pharmacy o Academic hospital o Drug company o Industry		104 (49.5) 70 (33.3) 19 (9.0) 16 (7.6) 1 (0.05)

IQR: Interquartile range.

Regarding pharmacists’ awareness towards CPD (Table 2), more than half of the participants were unfamiliar with the concept of CPD (*n* = 108, 51.7%), while a minority were very familiar (*n* = 8, 3.8%). Participants were seldom able to define CPD correctly (*n* = 17, 8.2%). Most participants believed CPD was applied worldwide (*n* = 156, 74.6%), and some thought it was undertaken by Jordanian pharmacists (*n* = 67, 32.1%). Some pharmacists thought there was a legislation that mandates Jordanian pharmacists to undertake CPD (*n* = 60, 28.7%).

**Table 2.** Assessment of pharmacists’ awareness towards CPD (*n* = 210).

Question	*n* (%)
How familiar are you with the concept of CPD? o Unfamiliar o Slightly familiar o Moderately familiar o Very familiar	108 (51.7) 59 (28.2) 33 (15.8) 8 (3.8)
What is CPD? o Correct answer o Incorrect answer/I don't know	17 (8.2) 193 (91.9)
Do you think CPD is undertaken by pharmacists worldwide? o Yes o No	156 (74.6) 53 (25.4)
Do you think CPD is undertaken by pharmacists in Jordan? o Yes o No	67 (32.1) 142 (67.9)
Is there any legislation to implement CPD within pharmacy sector in Jordan? o Yes o No	60 (28.7) 148 (71.3)

**Figure 1.**
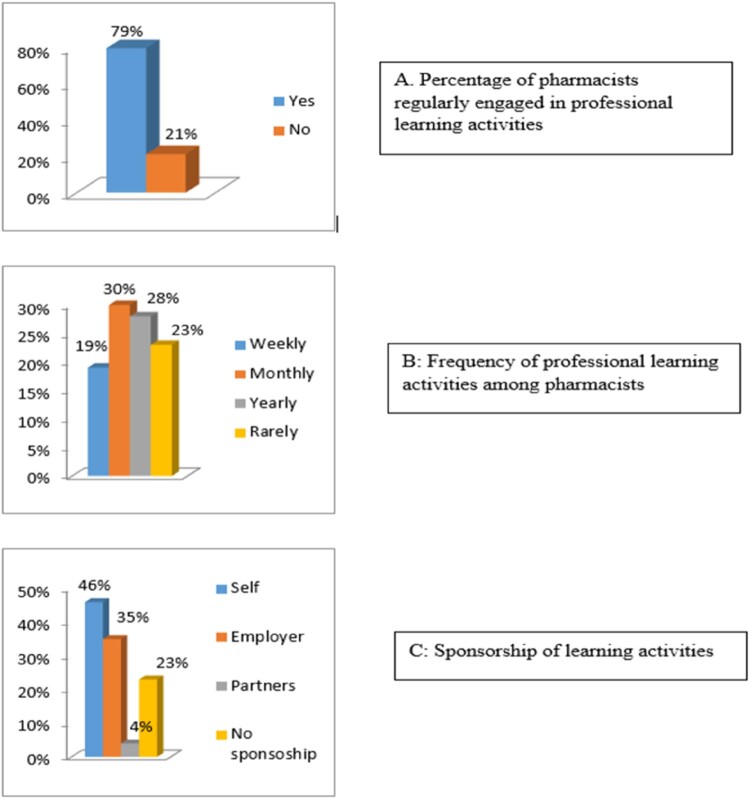
Pharmacists recent engagement in CPD activities (*n* = 210): (A) percentage of pharmacists regularly engaged in professional learning activities; (B) frequency of professional learning activities among pharmacists; (C) sponsorship of learning activities.

Looking at the current involvement of Jordanian pharmacists in professional learning activities, 80% of participants were engaged in some form of learning activities at various frequencies; 19% weekly, 30.5% monthly, 27.6% yearly and 22.9% rarely. Almost half of the participants (45.7%) were engaged in self-sponsored professional learning activities, and only 35.2% of participants were engaged in professional learning activities sponsored by their employers (Figure 1).

**Figure 2.**
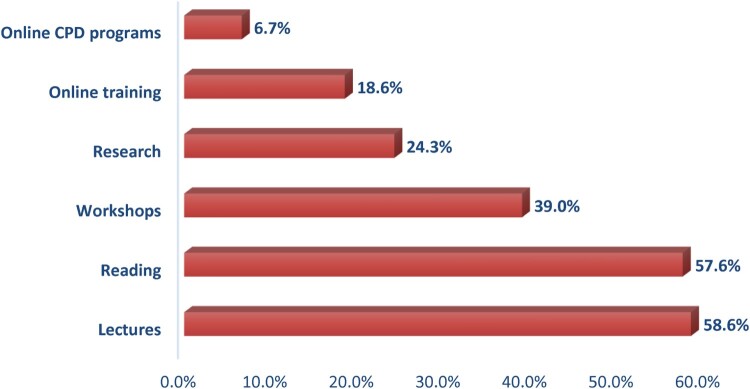
Types of learning activities pharmacists do usually use for their continuous professional learning process (*n* = 210).

**Figure 3.**
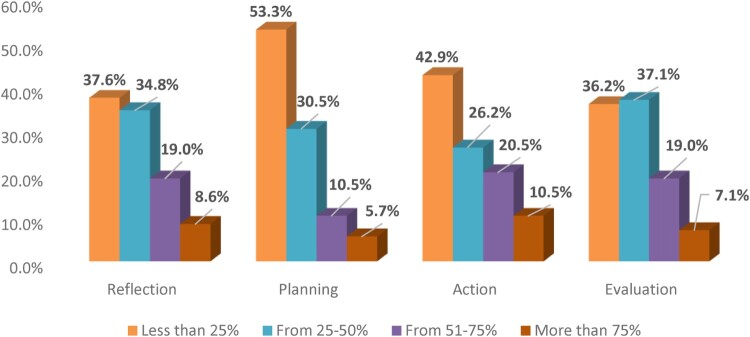
The extent to which pharmacists accomplish the four steps of CPD: reflection, planning, action and evaluation throughout their professional learning activities. Performance evaluated according to the frequency of accomplishing these steps (*n* = 210).

Participating pharmacists were engaged in various types of learning activities and were allowed to choose more than one option at a time; attending live lectures was the most used learning method with a percentage of 58.6%, followed by reading with a percentage of 57.6%, then engagement in interactive workshops39%, online training courses 18.6%, involvement in research 14.3%, and the least was using online CPD courses 6.2% (Figure 2). When asked about their performance and the extent to which they apply the four steps of CPD cycle (reflection, planning, action and evaluation) throughout their professional learning methods. Only 8.6% of the pharmacists claimed that they reflected their needs more than 75% of the time. Regarding pre-planning for their professional learning activities, only 5.7% planned for their learning activities more than 75% of the time. Relating to the extent to which participants accomplished their professional learning plans, 10.5% seemed to accomplish their plans for professional learning activities more than 75% of the time. With respect to evaluation of their professional learning processes after accomplishing them, 7.2% evaluated their professional learning p more than 75% of the time (Figure 3).

Majority of participants strongly/agreed that CPD should be mandatory to ensure compliance (*n* = 167, 80.6%), that CPD should be practiced under a legal framework (*n* = 170, 81.3%), and that it should be regulated by the MOH for Pharmacy practitioners (*n* = 167, 79.5%). Also, the majority of participants (*n* = 184, 88%) strongly agreed/agreed that undertaking CPD should be a lifelong learning process to help pharmacists stay competent and professional.

When asked about their confidence level in their ability/skill to apply the stages of CPD, 81% of them strongly agreed/agreed to be confident in their ability to identify their professional and work-related learning needs, 74.3% strongly agreed/agreed to be confident in their ability to plan for those needs, and 78.1% strongly agreed/agreed to be confident in their ability to evaluate the impact and outcomes of their learning after a professional learning activity (Table 3).

**Table 3.** Pharmacists’ views on the implementation of CPD activities (*n* = 210).

Statements	Strongly agree/agree *n* (%)
CPD should be mandatory to ensure compliance	167 (80.7)
To be able to be enforced well, CPD program should be inserted in a legal framework	170 (81.3)
Ministry of Health(MOH)should regulate CPD for pharmacy practitioners in Jordan	167 (79.5)
CPD providers for pharmacy practitioners should be regulated by JPA before they roll out CPD programs	144 (68.6)
Undertaking CPD should be a lifelong learning process to help pharmacists stay competent and professional	184 (88.0)
I am confident in my ability/skills to identify my learning needs related to my work or professional practice	170 (81.0)
I am confident in my ability/skills to plan my learning and professional development	156 (74.3)
I am confident in my ability/skills to evaluate the impact or outcomes of my learning	164 (78.1)

With respect to the anticipated benefits of a CPD program if it was implemented, the vast majority of participants strongly agreed/ agreed that CPD will keep their medical knowledge and professional skills updated (*n* = 195, 92.9%), improve their performance in their current role (*n* = 190, 90.5%), make them more confident and able to provide a better care for their patients (*n* = 191, 91%), improve the reputation of pharmacy profession among the public (*n* = 186, 88.6%) and improve the profession's reputation among other health-care professionals (*n* = 184, 87.6%). The majority also strongly agreed/agreed that it will enhance their career prospects (*n* = 182, 86.7%) and the interaction and exchange of knowledge with their colleagues (*n* = 187, 89%) (Table 4).

**Table 4.** Pharmacists’ perception towards the benefits, motivators and barriers of CPD implementation (*n = *210).

Statements	Strongly agree/agree *n* (%)
**Benefits of CPD activities implementation**	
Keeps my professional knowledge and skills updated	195 (92.9)
Improves my performance in my current role	190 (90.5)
Makes me more confident, competent and able to provide a higher-quality patient care	191 (91.0)
Enhances the reputation of the profession among the public	186 (88.6)
Enhances the reputation of the profession among other health-care providers	184 (87.6)
Enhances my career prospects	182 (86.7)
Enhances interaction and exchange of knowledge with coworkers	187 (89.0)
**Barriers for the implementation of CPD**	
Life and Work Commitments	163 (78.0)
Lack of Time	154 (73.7)
Difficulty in commuting to the CPD activity	121 (57.9)
Lack of information about the available activities	155 (74.2)
Lack of funding or reimbursement	144 (69.2)
Inadequate staffing or time-off work	147 (70.3)
Lack of policy guidelines for CPD	152 (72.7)
**Motivators for the implementation of CPD**	
CPD activities close to work place	172 (82.3)
CPD activities with flexible times and dates	177 (84.7)
CPD activities with wide range of topics	182 (87.1)
CPD methods available online	174 (83.3)
Employer support (time-off, funding)	179 (85.6)

Concerning the factors that would motivate them to undertake CPD, participants strongly agreed/agreed for important factors that would enhance their participation. Offering CPD activities close to their work place (*n* = 172, 82.3%), having CPD activities with flexible range of times and dates (*n* = 177, 84.7%), CPD activities with diverse and comprehensive topics that meet pharmacists’ practice needs (*n* = 182, 87.1%), availability of online or technology-based CPD activities (*n* = 174, 83.3%), and employer's support by offering time off and/or funding (*n* = 179, 85.6%) (Table 4).

When asked about the barriers that may hinder the implementation of CPD, participants felt the biggest barrier is life and work commitments (*n* = 163, 78%), followed by lack of information about the available CPD activities available (*n* = 155,74.2%), lack of time (*n* = 154, 73.7%), lack of policy guidelines for CPD (*n* = 152, 72.7%), inadequate staffing (*n* = 147, 70.3%) and lack of funding and reimbursement (*n* = 144, 69.2%), and, to a less extent, difficulty travelling to the CPD activity (*n* = 121, 57.9%) (Table 4).

## Discussion

Continuing Professional Development, endorsed by the World Health Organization (WHO) and the FIP, is a self-directed, outcome-oriented, lifelong learning approach designed to ensure pharmacists remain competent by continuously updating their knowledge, enhancing their skills, and maintaining patient-centred practices (FIP, 2002; WHO, 1998). Over the past decade, CPD has been successfully adopted by several countries, including Canada, the UK, and the USA, and has proven to be highly effective in pharmacy practice. It has significantly improved pharmacists’ skills and attitudes, which in turn positively influences practice outcomes (Fjortoft & Schwartz, 2003; McConnell et al., 2012; Rouse, 2004b). However, CPD remains a relatively new concept in Jordan's pharmacy landscape, highlighting the need for its introduction and widespread adoption within the profession. To facilitate this, it is essential to first assess the awareness and perceptions of Jordanian pharmacists regarding CPD. Therefore, the primary aim of this study is to evaluate the current status of CPD in Jordan, while also exploring the motivators that may encourage pharmacists to engage in CPD activities and identifying the barriers that may prevent their participation.

Most participants in this study demonstrated a low level of awareness to CPD concept, as evidenced by their inability to define it and their uncertainty regarding its implementation in Jordan. It was unsurprising to find that a large proportion of participants were unfamiliar with CPD, and struggled to define it accurately, given that CPD is a new concept to the pharmaceutical field in Jordan and has not been previously applied or promoted. This finding mirrors similar situations observed in countries that transitioned from CE to CPD, where pharmacists exhibited low awareness of CPD and required training and educational sessions on how to apply CPD cycle elements (Dopp et al., 2010; Haughey et al., 2007; Tofade et al., 2010).

Although the vast majority of participants believed that CPD is practiced globally but not in Jordan, some held misconception regarding legislation mandating CPD for pharmacists in Jordan, which has not been activated or implemented. Unfortunately, this is not the reality; Jordan currently lacks legislation in this regard. This finding underscores the lack of awareness among Jordanian pharmacists for CPD awareness. This finding is consistent with research conducted by Alnahar et al. (2024), which indicated that despite positive attitudes toward CPD, barriers such as time, cost, and logistical constraints remain significant obstacles for Middle Eastern pharmacists (Alnahar et al., 2024).

Participants were observed to engage in various forms of professional learning processes and activities, such as reading, attending workshops, lectures, using online resources and occasionally participating in research. These learning habits closely resemble those reported by British Pharmacists (Attewell et al., 2005; Mottram et al., 2002). However, the frequency of engagement in learning activities among Jordanian participants, primarily on weekly or monthly basis, appears to be lower than that reported by British pharmacists who indicated weekly reading habits (Attewell et al., 2006). This low frequency can be attributed to multiple factors, as identified in our study, including the absence of legislation, lack of funding and limited availability of CPD activities.

Some pharmacists were involved in research, while others engaged in online training and small number participated in online CPD courses. This may be due to the limited availability of online resources and the unfamiliarity with the CPD approach as a sustainable learning tool. Hjazeen and colleagues found similar issues in Jordan, where community pharmacists expressed positive views on CPD but faced substantial barriers such as limited access to resources and logistical constraints (Hjazeen, 2023).

Participants rarely implemented elements of the CPD cycle – reflection, planning, action and evaluation – in their professional learning activities. This could be attributed to a scarcity of resources and limited awareness to CPD concept and how to implement it. These findings closely consistent with those reported in Great Britain, where the extent of CPD cycle accomplishment was measured. There, pharmacists show poor understanding and application of CPD cycle elements, particularly in reflection and documentation (Attewell et al., 2006). Similarly, Darwish et al. (2023) highlighted that pharmacists in Middle East face challenges in implementing the CPD cycle, with barriers such as job restrictions and lack of time significantly impeding engagement (Darwish et al., 2023).

Despite the reported low level of awareness, the majority of the participants agreed that CPD should be mandatory, incorporated into a legal framework and should be regulated by the MOH in order to assure compliance. This was also recommended by other accordant studies (Attewell et al., 2006; Driesen et al., 2007; Haughey et al., 2007; Mottram et al., 2002; Swainson & Silcock, 2004; USAID, 2013). Such enthusiasm provides hope for fruitful CPD application in the future.

Although the CPD concept is new to Jordanian pharmacists, many participants surprisingly reported confidence in their ability to identify their needs, plan learning activities and to evaluate their professional learning. However, these reports may not be accurate and could be explained by lack of awareness to CPD cycle elements their implementation.

Benefits of the CPD program were anticipated to be keeping knowledge and professional skills up to date, improving performance, boosting confidence in providing superior patient care, and enhancing the general reputation of the pharmacy profession among the public and other health-care providers. These benefits closely mirror those observed in other countries following the implementation of CPD, which have been shown to improve patient care, professional knowledge, skills, attitudes and values (McConnell et al., 2010; Tofade et al., 2013). Such a belief holds significant promise for the ease of CPD application in the future.

Motivators for undertaking CPD were identified as proximity to workplace, frequent learning activities available at various times, a diverse range of topics that meet pharmacists’ learning needs, availability of CPD methods online, a better understanding of which work activities are suitable for CPD, additional educational training on the CPD concept and its application, and employer support, including time off and reimbursement. These findings are consistent with those of a literature review of British pharmacists (Donyai et al., 2011). Such motivators should be considered by regulatory bodies when implementing CPD programs.

Barriers to undertaking CPD primarily included a lack of time and conflicting life and work commitments, consistent with findings from other studies (Bell et al., 2001; Donyai et al., 2011), which identify similar obstacles such as time constraints, financial costs and resource issues, understanding of CPD, facilitation and support for CPD, system constraints, and technical problems (Donyai et al., 2011; McConnell et al., 2012). Additional barriers included difficulty travelling to activity site. This is particularly relevant for pharmacists living outside Amman, where most of the medical professional learning activities occur. As the capital city of Jordan, Amman has the highest density of pharmacists and drug companies, which sponsor the majority of the ongoing medical professional education activities.

A significant barrier is the lack of information about learning activities, compounded by Jordan's absence of a regulatory body to oversee continuing education activities or certified CPD providers to organised, and properly publicise structured learning activities by media and social media. Furthermore, the lack of funding, reimbursement, is a crucial yet difficult barrier to overcome. Pharmacists are unlikely to be motivated to undertake CPD if it represents a financial cost, an issue that regulatory bodies need to consider before mandating CPD. Additionally, inadequate staffing to cover for pharmacists who leave work to participate in learning activities exacerbates the financial cost barrier, as having more pharmacists for coverage incurs additional expenses. The lack of policy guidelines for CPD is an expected and reasonable barrier, since CPD is neither introduced to pharmacists in Jordan nor mandated by the government.

This study is the first to evaluate the current status, views, motivators, and barriers related to CPD among Jordanian pharmacists. However, it has several limitations. First, the study relied on a self-administered questionnaire, which may overestimate pharmacists’ awareness of CPD. Second, the sample was limited to pharmacists located in Amman, the capital city, which may not represent the views and practices of pharmacists in other parts of the country. Future studies could aim to include a broader geographic sample and explore other regions of Jordan.

Despite these limitations, the findings of this study provide valuable insights that can inform the future implementation of CPD in Jordan. It is clear that while there is enthusiasm for CPD, significant barriers exist, including a lack of legislation, funding, and access to CPD opportunities. Policymakers and regulatory bodies must work to address these barriers and ensure that CPD becomes an integral part of the professional development of pharmacists in Jordan.

## Conclusion

Following this study, a clearer picture has emerged regarding the awareness, perception, and attitudes of Jordanian pharmacists towards CPD. Our findings could serve as an eye-opener for regulatory bodies before mandating CPD. It is crucial to raise awareness about the CPD concept among Jordanian pharmacists through regular campaigns, and media and social media engagements. Furthermore, considering all motivators for CPD identified in our study could encourage pharmacists to undertake CPD and maintain a CPD portfolio.
